# Oral Health Knowledge, Attitude, And Practices Among Residents Of Al-kharj, Saudi Arabia: A Cross-sectional Study

**DOI:** 10.12688/f1000research.172953.1

**Published:** 2025-11-26

**Authors:** Sultan Almalki, Abdulrahman Mohammed Alaskar, Inderjit Murugendrappa Gowdar, Abdullah Saad Alqahtani, Khalid Gufran, Bhuvaneshwari Nadar, Usha GV

**Affiliations:** 1 Preventive Dental Sciences, Prince Sattam bin Abdulaziz University College of Dentistry, Al Kharj, Riyadh Province, 11942, Saudi Arabia; 2 Public Health Dentistry, Terna Dental College and Hospital, Nerul, Navi Mumbai, Maharashtra, 400706, India; 3 Public Health Dentistry, Bapuji Dental College and Hospital, Davangere, Karnataka, 577004, India

**Keywords:** Oral Health, Knowledge, Attitude, Behaviour, Preventive Dental Visits

## Abstract

**Background and Objective:**

Several studies highlight the widespread prevalence of dental health problems within the Saudi population. To effectively develop public health strategies, it is vital to assess oral health knowledge, attitudes, and behaviours at a population level. This study aimed to examine these three domains among adults aged 18 and older residing in Al-Kharj, Saudi Arabia.

**Methods:**

A cross-sectional survey was conducted in 2024, involving a sample of adult visitors at shopping malls and public parks in Al-Kharj. Data were gathered using a validated, self-administered, structured questionnaire addressing knowledge, attitudes, and behaviours related to oral health.

**Results:**

The study included 367 participants aged 18 years and above. Among them, 90.2% were aware of the link between tobacco use and oral cancer, and 89.1% held favourable views regarding the use of fluoridated toothpaste to prevent cavities. However, only 55.6% brushed their teeth twice daily, and just 44.7% routinely visited a dentist. Women exhibited significantly better knowledge than men (p = 0.0001), older individuals had greater knowledge than younger ones (p = 0.004), and participants with a monthly income above 10,000 SAR scored higher on oral health knowledge (p = 0.003).

**Conclusion:**

Although many respondents demonstrated good oral health knowledge, fewer than half maintained positive attitudes and practices necessary for proper oral care.

## Introduction

Teeth are an integral part of the human body and reflect each individual’s health history and lifestyle. Oral health is a window into overall well-being, often revealing systemic health issues. It is defined as the absence of diseases affecting the teeth, gums, and supporting tissues. Oral hygiene practices play a crucial role in promoting dental and general health, improving appearance, and enhancing self-esteem. ^1^

In recent decades, there has been a surge in chronic conditions, including dental caries, cardiovascular diseases, and cancers, all of which exert social, economic, and political pressure. Oral diseases, often lifelong and preventable, cause pain and financial burden to individuals and healthcare systems alike. ^2^

The World Health Organization (WHO) reports that around 3.5 billion people globally suffer from permanent dental decay. ^3^ Despite advancements, oral diseases remain a global concern due to the inconsistent adoption of preventive practices such as daily brushing and regular dental visits. In nations lacking established oral health initiatives, dental caries continues to be widespread. ^4, 5^

Numerous studies within Saudi Arabia have reported a high prevalence of oral diseases. ^6^ A systematic review of research from 1988 to 2010 revealed that 62%–84% of children under six suffered from caries in primary teeth, while 58%–94% were affected in permanent teeth. ^7^ A Riyadh-based study showed only 6.3% of 12–14-year-olds were caries-free. ^8^

Oral health outcomes are influenced by both proximal factors (such as behaviour and access to dental care) and distal factors (such as socioeconomic status and home environment). ^9^ International studies among university students have shown poor oral hygiene practices, regardless of country income level. ^10^ Research in Nigeria and India also reported inadequate oral health knowledge and behaviour among healthcare students, with females generally outperforming males. ^11, 12^

Barriers to regular dental care, including limited awareness, are associated with modifiable behaviour’s. A study from Qatar found a direct link between dental knowledge and healthy oral habits like brushing and dentist visits. ^13^ Studies across Arab nations have mainly focused on student populations, often comparing dental students without considering the general adult population. ^14– 17^ Understanding public knowledge and behaviour is essential to improving national health outcomes.

Oral health is often compromised by lack of knowledge or disregard for hygiene practices. Educated individuals who apply what they know tend to achieve better dental health. Acknowledging personal responsibility for oral care often leads to improved self-care. Since dentists are key in educating communities, evaluating knowledge, attitudes, and behaviour’s at the population level is vital. This study investigates these factors among adults in Al-Kharj and examines the associations between demographic factors and oral health practices.

## Materials and method

This study was designed, conducted, and reported in accordance with the STROBE (Strengthening the Reporting of Observational Studies in Epidemiology) guidelines.

**Study Design:** A descriptive cross-sectional survey was undertaken, involving 367 adult residents of Al-Kharj city. Data collection began in June 2024 and continued over a three-month period.

**Participants:** The study targeted adults who visited shopping malls, as these venues provided access to a diverse and representative sample of the city’s population across various age groups.

**Sample Size:** The required sample size was determined using the formula N = 4pq/l ^2^. Drawing on pilot data that estimated the prevalence of poor oral health knowledge at 35.36% and allowing for a 5% margin of error, the final sample size was calculated as 367 participants.

### Inclusion criteria

Participants were eligible if they were 18 years or older, had resided in Al-Kharj city for at least one year, and were able to complete an online questionnaire via Google Forms. Individuals were approached in shopping malls and other public areas and were invited to participate if they were available and willing.

### Sampling technique

A multistage stratified random sampling method was employed to recruit participants from shopping malls and public parks across Al-Kharj City. The city was segmented into four geographic zones: East, West, South, and North. Within each zone, at least one shopping mall and two public parks were randomly chosen using a random number generator. This process resulted in the selection of four malls and eight parks overall. From each selected location, 92 individuals were enrolled in the study, except for one zone where 91 participants were recruited. In cases where the number of available participants was insufficient at a site, additional visits were made on subsequent days to fulfill the required sample. Care was taken to include only one individual per family or social group to avoid sampling bias.

**Data collection:** Participants were asked to complete a comprehensive, validated, self-administered, structured questionnaire consisting of 35 closed-ended items available in both Arabic and English. The questionnaire was divided into four sections:

1. **Sociodemographic Information (11 items):** Covered variables such as age, gender, education, occupation, household income, nationality, housing ownership, general health status, smoking, alcohol use, and frequency of dental visits.
2. **Oral Health Knowledge (11 items):** Multiple-choice questions focused on oral hygiene practices. A score of 1 was assigned for each correct answer and 0 for incorrect or “don’t know” responses.
3. **Attitudes Toward Oral Health (8 items):** Evaluated using a five-point Likert scale ranging from “strongly agree” to “strongly disagree.”
4. **Oral Health Behaviour’s (7 items):** Assessed participants’ practices using response categories: never, seldom, occasionally, very often, and always.

To ensure linguistic accuracy, the questionnaire was initially translated into Arabic and then back-translated into English by a bilingual expert using the back-translation method. This process helped achieve linguistic equivalence. Content validity was evaluated by two subject matter experts—one specializing in Public Health Dentistry and the other in Periodontics—resulting in a Content Validity Index (CVI) of 0.82, indicating a high level of agreement.

A pilot study was conducted to test the reliability of the instrument. The questionnaire was administered to 10 randomly selected individuals at a shopping mall. After three days, the same participants were asked to complete the questionnaire again via WhatsApp. The internal consistency of the tool was measured using Cronbach’s alpha, yielding a value of 0.80, which signifies good reliability.

The final version of the questionnaire was self-administered under the supervision of two trained and calibrated investigators, ensuring consistency and accuracy in data collection.

### Ethics and consent

The study had obtained ethical clearance from Institutional Review Board and Ethical Approval Committee from Prince Sattam bin Abdulaziz University, Al Kharj (SCBR-438/2025). All participants received an explanation of the study and gave voluntary written informed consent before completing the self-administered questionnaire.

### Statistical analysis

The data were analyzed by using statistical analysis software (SPSS version 25, IBM, Armonk, NY, USA). A p-value of <0.05 was considered statistically significant. Descriptive statistics were calculated by using means and standard deviations for continuous variables and frequencies and percentages for categorical variables. The chi-square test was used to assess associations between categorical variables. A knowledge score was calculated from the 11 knowledge questions; a score of 1 was given for correct answers and 0 for incorrect or “I don’t know” answers. By adding the scores of all knowledge questions, we calculated a total knowledge score for each participant, ranging from 0 to 11. Higher scores indicated better knowledge. Linear regression was used to determine predictors of adults’ knowledge scores. The predictors assessed were gender, age, education, and monthly income; the outcome was the knowledge score. A forward stepwise technique was used to select variables for inclusion in the model, with an entry level p-value of 0.05 and a removal level p-value of 0.1.

## Results

Table 1 presents the demographic characteristics of the study population. The study included 367 adults, comprising 177 males and 190 females, aged between 18 and 69 years. Female participants made up a significantly larger portion of the sample compared to males. The largest age group represented was 25–34 years (38.4%), while participants aged 65 years and older formed the smallest group (1.1%). Most participants (89.6%) were Saudi nationals, and 61.6% were married. Regarding education, 51.0% held a university degree, 30.0% had completed high school, and 9.5% had finished primary school. In terms of income, 30.0% earned between 20,000 and 40,000 Saudi Riyals, while 5.4% reported incomes exceeding 100,000 Saudi Riyals. Approximately 68.6% of participants reported no systemic health conditions; diabetes mellitus was present in 14.7% of participants, followed by hypertension in 9.8%. The majority (80.1%) were non-smokers, and none reported alcohol consumption.

**Table 1.** Demographic details of study participants.

Variables	N (Total = 367)	Percentage
Gender		
Male	177	48.2
Female	190	51.8
Age groups		
18-24 yrs	51	13.9
25-34 yrs	141	38.4
35-44 yrs	100	27.2
45-54 yrs	57	15.5
55-64 yrs	14	3.8
65 and above yrs	4	1.1
Citizenship		
Citizens	329	89.6
Non-citizen	38	10.4
Marital status		
Unmarried	141	38.4
Married	226	61.6
Education		
Primary school	35	9.5
Intermediate	35	9.5
High school	110	30.0
College	187	51.0
Ownership		
Rented	108	29.4
Owner	259	70.6
Family income		
<20000 SR	73	19.9
20000-40000 SR	110	30.0
40000-60000 SR	91	24.8
60000-80000 SR	38	10.4
80000-100000 SR	35	9.5
>100000 SR	20	5.4
General health		
Diabetes Mellitus	54	14.7
Hypertension	36	9.8
Asthma	5	1.5
Anaemia	20	5.4
Healthy	252	68.6
Smoking status		
No	294	80.1
Yes	73	19.9
Alcohol consumption		
No	367	100
Yes	0	

Table 2 details the distribution of oral health knowledge, attitudes, and behaviour’s among participants. Most participants demonstrated fair to good knowledge about oral health. Notably, 90.2% were aware that tobacco use can cause oral cancer, but only 28.6% recognized the importance of treating primary teeth. A large majority (89.1%) had a positive attitude towards the use of fluoridated toothpaste for preventing dental caries, while 56.2% showed a negative attitude towards the role of tooth brushing in preventing gum diseases. Furthermore, 65.7% believed that dentists focus not only on treatment but also on the prevention of oral diseases. However, only 44.5% exhibited good behaviour in valuing dental health equally with general health. Just 55.6% brushed their teeth twice a day, and 44.7% attended regular dental check-ups.

**Table 2.** Distribution of oral health knowledge, attitude and behaviour across study population.

Questionnaire	Response	
*Knowledge*	**Correct**	**Incorrect**
There are two sets of teeth during lifetime	270 (73.6)	97 (26.4)
Tooth infection causes gum bleeding	234 (63.8)	133 (36.2)
Replacement of missing tooth improves oral hygiene	293 (79.8)	74 (20.2)
The tooth decay of milk teeth need to be treated	262 (71.4)	105 (28.6)
Bacteria is one of the reasons to cause gum problems	298 (81.2)	69 (18.8)
Soft drinks affect the teeth adversely	330 (89.9)	37 (10.1)
Loss of teeth can interfere with speech	324 (88.3)	43 (11.7)
Irregularly placed teeth can be moved into correct position by dental treatment	293 (79.8)	74 (20.2)
Decayed teeth can affect the appearance of a person	327 (89.1)	40 (10.9)
Tobacco chewing, or smoking can cause oral cancer	331 (90.2)	36 (9.8)
White patches on teeth are called dental plaque	250 (68.1)	117 (31.9)
*Attitude*	**Positive**	**Negative**
Brushing my teeth twice a day improves oral hygiene	249 (67.8)	118 (32.2)
Keeping my teeth clean and healthy is beneficial to your health	285 (77.7)	82 (22.3)
I believe improper brushing leads to gum disease	322 (87.7)	45 (12.3)
Eating sticky sweet is major reason for tooth decay	284 (77.4)	83 (22.6)
To prevent tooth decay everyone should brush with fluoridated tooth paste	337 (89.1)	40 (10.9)
Dentists care only about treatment & not prevention	241 (65.7)	126 (34.3)
No matter how carefully I brush, gum bleeding is inevitable	167 (43.8)	206 (56.2)
Only professional tooth cleaning prevents gum bleeding	167 (55.1)	202 (44.9)
*Behaviour*	**Adequate**	**Inadequate**
I give importance to my teeth as much as any part of my body	163 (44.5)	204 (55.5)
I brush my tooth twice daily	204 (55.6)	163 (44.4)
I use teeth to open cap of bottled drink	81 (22.1)	286 (77.9)
I experience toothache while chewing food	162 (44.1)	205 (55.9)
I have bleeding gums during brushing	122 (33.3)	245 (66.7)
I go for routine dental check-up	164 (44.7)	203 (55.3)

Table 3 illustrates the association between oral health knowledge and gender. Males demonstrated better oral health knowledge compared to females. All female participants and 40.7% of male participants displayed poor knowledge regarding the importance of treating primary teeth.

**Table 3.** Relationship between oral health knowledge and gender.

Item	Male (N, %)	Female (N, %)	X ^2^ value	p-value
There are two sets of teeth during lifetime			122.827	0.000
Yes	77 (43.5)	93 (48.9)		
No	100 (56.5)	97 (51.1)		
Tooth infection causes gum bleeding			194.322	0.000
Yes	50 (28.3)	133 (70)		
No	127 (71.7)	57 (30)		
Replacement of missing tooth improves oral hygiene			86.348	0.000
Yes	89 (50.3)	116 (61.1)		
No	88 (49.7)	74 (38.9)		
The tooth decay of milk teeth need not be treated			157.883	0.000
Yes	105 (59.3)	0		
No	72 (40.7)	190 (100)		
Bacteria is one of the reasons to cause gum problems			79.162	0.000
Yes	65 (36.8)	121 (63.7)		
No	112 (63.2)	69 (36.3)		
Soft drinks affect the teeth adversely			38.333	0.000
Yes	100 (56.5)	153 (80.5)		
No	70 (43.5)	37 (19.5)		
Loss of teeth can interfere with speech			45.374	0.000
Yes	68 (38.4)	147 (77.3)		
No	109 (61.6)	43 (22.7)		
Irregularly placed teeth can be moved into correct position by dental treatment			86.348	0.000
Yes	96 (54.2)	116 (61.1)		
No	81 (45.8)	74 (38.9)		
Decayed teeth can affect the appearance of a person			41.821	0.000
Yes	90 (50.8)	150 (78.9)		
No	87 (49.2)	40 (21.1)		
Tobacco chewing, or smoking can cause oral cancer			37.184	0.000
Yes	120 (67.8)	130 (68.4)		
No	57 (32.2)	60 (31.6)		
White patches on teeth are called dental plaque			160.004	0.000
Yes	50 (28.3)	117 (61.6)		
No	127 (71.7)	73 (38.4)		

Table 4 presents the relationship between oral health attitude and gender. A majority (80.7%) of males strongly agreed on the importance of brushing twice daily, and all male participants strongly agreed that sticky sweets contribute to dental decay. However, 90.4% of males exhibited a negative attitude toward the dentist’s role in preventing dental diseases. Additionally, 90.9% of males strongly agreed that bleeding gums can be prevented through professional dental cleaning.

**Table 4.** Relationship between attitude towards oral health and gender.

Item	Response	N (367)	Male (177)	Female (190)	X ^2^ value	p-value
Brushing my teeth twice a day improves oral hygiene	Strongly agree	262 (71.4)	98 (55.3)	164 (86.3)	276.206	0.000
	Agree	105 (28.6)	79 (44.7)	26 (12.7)		
	Neutral	0	0	0		
	Disagree	0	0	0		
	Strongly disagree	0	0	0		
Keeping my teeth clean and healthy is beneficial to your health	Strongly agree	208 (45.5)	100 (56.4)	108 (56.8)	172.346	0.000
	Agree	159 (32.1)	77 (43.6)	82 (43.2)		
	Neutral	0	0			
	Disagree	0	0			
	Strongly disagree					
I believe Improper brushing leads to gum disease	Strongly agree	52 (14.1)	30 (16.9)	22 (11.6)	288.630	0.000
	Agree	221 (60.2)	98 (55.3)	123 (64.7)		
	Neutral	25 (6.8)	25 (14.1)	0		
	Disagree	69 (18.8)	24 (13.5)	45 (23.7)		
	Strongly disagree	0	0	0		
Eating sticky sweet is major reason for tooth decay	Strongly agree	197 (53.7)	90 (50.8)	107 (56.3)	99.918	0.000
	Agree	170 (46.3)	87 (49.2)	83 (43.7)		
	Neutral	0	0	0		
	Disagree	0	0	0		
	Strongly disagree	0	0	0		
To prevent tooth decay everyone should brush with fluoridated tooth paste	Strongly agree	106 (28.8)	0	106 (55.8)	174.462	0.000
	Agree	151 (41.2)	67 (37.9)	84 (44.2)		
	Neutral	110 (30)	110 (62.1)	0		
	Disagree	0	0	0		
	Strongly disagree	0	0	0		
Dentists care only about treatment & not prevention	Strongly agree	160	160 (90.4)	0	313.204	0.000
	Agree	81	17 (9.6)	64 (33.7)		
	Neutral	41	0	41 (21.6)		
	Disagree	43	0	43 (22.6)		
	Strongly disagree	42	0	42 (22.1)		
No matter how carefully I brush, gum bleeding is inevitable	Strongly agree	79 (21.6)	79 (44.6)	0	311.324	0.000
	Agree	82 (22.3)	82 (46.3)	0		
	Neutral	122 (33.3)	16 (9)	106 (55.8)		
	Disagree	43 (11.7)	0	43 (22.6)		
	Strongly disagree	41 (11.1)	0	41 (21.6)		
Only professional tooth cleaning prevents gum bleeding	Strongly agree	161 (43.8)	161 (90.9)	0	327.927	0.000
	Agree	41 (11.2)	16 (9.1)	25 (13.1)		
	Neutral	122 (33.3)	0	122 (64.2)		
	Disagree	43 (11.7)	0	43 (22.6)		
	Strongly disagree	0	0	0		

Table 5 outlines the relationship between oral health behaviour and gender. Most participants did not view dental health as equally important as overall body health. Only 43% of female participants brushed their teeth twice daily. Approximately 7.4% of males reported using their teeth to open bottle caps, and 23.2% of males experienced tooth pain while chewing food. A majority of males (68.9%) reported bleeding gums during brushing. In comparison to males, a greater proportion of females (64.7%) attended routine dental check-ups.

**Table 5.** Relationship between oral health behavior and gender.

Item	Response	N (367)	Male (177)	Female (190)	X ^2^ value	p-value
I give importance to my teeth as much as any part of my body	Always	0	0	0	276.667	0.000
	Very often	81 (22)	0	81 (42.6)		
	Often	82 (22.3)	0	82 (43.1)		
	Seldom	164 (44.7)	137 (77.4)	27 (7.4)		
	Never	40 (11)	40 (22.6)	0		
I brush my tooth twice daily	Always	108 (29.4)	0	108 (56.8)	317.346	0.000
	Very often	41 (11.1)	0	41 (21.6)		
	Often	55 (15)	14 (7.9)	41 (21.6)		
	Seldom	123 (33.5)	123 (69.5)	0		
	Never	40 (11)	40 (22.6)	0		
I use teeth to open cap of bottled drink	Always	0	0	0	320.483	0.593
	Very often	0	0	0		
	Often	0	0	0		
	Seldom	13 (3.5)	13 (7.4)	0		
	Never	354 (96.2)	164 (92.6)	190 (100)		
I experience toothache while chewing food	Always	0	0	0	274.005	0.000
	Very often	0	0	0		
	Often	69 (18.8)	41 (23.2)	28 (14.8)		
	Seldom	218 (59.4)	136 (76.8)	82 (43.1)		
	Never	80 (21.8)	0	80 (42.1)		
I have bleeding gums during brushing	Always	55 (15)	55 (31.1)	0	245.221	0.000
	Very often	122 (33.2)	122 (68.9)	0		
	Often	0	0	0		
	Seldom	122 (33.2)	0	122 (64.2)		
	Never	68 (18.5)	0	68 (35.8)		
I go for routine dental check-up	Always	41 (11.2)	0	41 (21.6)	296.264	0.000
	Very often	0	0	0		
	Often	123 (33.6)	0	123 (64.7)		
	Seldom	81 (22)	55 (31.1)	26 (13.7)		
	Never	122 (33.2)	122 (68.9)	0		

Table 6 identifies the predictors of oral health knowledge scores. Males demonstrated significantly better oral health knowledge compared to females (p = 0.0001). Older individuals exhibited greater knowledge than younger adults (p = 0.004). Additionally, participants with an income exceeding 10,000 SR showed higher levels of oral health knowledge (p = 0.003).

**Table 6.** Predictors of oral health knowledge, attitude and behavior.

Variables	Co-efficient	Standard error	95% confidence interval		p-value
			Lower	Upper	
Gender (ref = Female) Male	0.56	0.10	0.21	0.77	0.001
Age (<35 yrs) ≥ 35 yrs	0.38	0.14	0.17	0.65	0.004
Education level (less than degree) Degree	-0.47	0.11	-0.11	1.01	0.790
Family income (<10,000 SR) >10,000 SR	0.26	0.12	0.30	0.98	0.003

Socio-demographic factors were not significant predictors of behaviour and attitude about oral health among our participants.

## Discussion

This cross-sectional study investigated the oral health knowledge, attitudes, and behaviour’s of 367 adult residents of Al-Kharj, recruited from shopping malls. The findings revealed that female participants demonstrated significantly higher levels of oral health knowledge, more positive attitudes, and better behavioural practices compared to males. Specifically, women showed greater awareness regarding the causes of gum bleeding and the importance of tooth brushing in maintaining gingival health. This may be attributed to a heightened aesthetic awareness among females, which encourages more frequent dental visits and greater exposure to oral health information. ^18^

Oral health attitudes are influenced by a range of factors, including individual experiences, family and cultural traditions, religious beliefs, and life circumstances, all of which shape behavioural outcomes. ^19^ In the current study, women reported superior oral hygiene practices, particularly in terms of tooth brushing frequency and regular dental visits—findings consistent with prior research. ^20– 24^ These positive behaviour’s may be linked to a stronger concern for personal appearance, prompting women to seek dental advice and education more regularly. Routine dental visits are essential not only for disease prevention but also for reinforcing good hygiene habits and providing patient education. ^25– 27^

In this study, 77.7% of participants acknowledged that maintaining healthy teeth contributes to overall health. This aligns with results from other regions, including 81% in Manipur, 76% among Nepalese children, and 93% of Iranian children who recognized the same connection. ^28, 29^ Interestingly, the current study found no significant association between education level and oral hygiene knowledge or behaviour. While higher education is generally linked to improved oral health awareness, knowledge alone may not translate into practice. Factors such as socioeconomic status, cultural norms, and resource availability also play critical roles in shaping oral health behaviour’s.

Oral health concerns are closely related to individual attitudes. In our study, influences stemming from cultural, religious, and familial beliefs were apparent. Notably, 90% of male participants perceived that dentists focus primarily on treatment rather than prevention, reflecting a reactive approach to dental care, often sought only during pain or discomfort. This perception reduces opportunities for preventive care. Dentists, along with mass media, have the potential to significantly influence public behaviour’s by effectively communicating oral health information. ^30, 31^ Our results further showed that participants who received brushing technique demonstrations from their dentists exhibited improved oral hygiene knowledge.

This study has several strengths, including the use of a validated and pilot-tested questionnaire and a sample drawn from diverse community settings. However, it is important to note several limitations. The sample size was relatively small, and participants were recruited from public venues such as malls and parks, which may introduce social desirability bias. Additionally, time constraints in these settings may have affected the accuracy of responses. Nevertheless, data collection in these areas allowed access to a broad and varied segment of the population, providing real-time insights. Another limitation was the absence of clinical oral examinations, which could have provided objective measures of oral health. Including clinical assessments would have strengthened the analysis by correlating self-reported knowledge, attitudes, and behaviour’s with actual oral health status.

## Conclusion

This population-based study involving adults aged 18 years and older revealed that women demonstrated superior oral health knowledge and more favourable attitudes toward dental care, with statistically significant gender differences. Around two-thirds of participants reported brushing their teeth twice daily, with women showing a greater appreciation for the importance of primary teeth and regular dental checkups compared to men. Based on these findings, we recommend the introduction of evidence-based and targeted oral health awareness initiatives to promote improved hygiene practices among adults in this age group.

## Ethics and consent

The study had obtained ethical clearance from Institutional Review Board and Ethical Approval Committee from Prince Sattam bin Abdulaziz University, Al Kharj. The Committee on Research Ethics adhered to international guidelines for human subject protection, including the Declaration of Helsinki, The Belmont Report, and CIOMS guidelines. All participants received an explanation of the study and gave informed consent before completing the self-administered questionnaire. The consent form clearly outlined the survey’s purpose, the estimated time required to complete the questionnaire, and used simple, understandable language. Participants were informed that they could withdraw from the survey at any time, and that their personal information would be kept confidential.

## Data Availability

Figshare – Oral health knowledge, attitude, and practices among residents of Al-kharj, Saudi Arabia: a cross-sectional study. https://doi.org/10.6084/m9.figshare.30507323.v1. ^32^ This project contains following underlying data:
• study-data.xlsx Data are available under the terms of the Creative Commons Zero “No rights reserved” data waiver (CC BY 4.0 Public domain dedication). Figshare - Oral health knowledge, attitude, and practices among residents of Al-kharj, Saudi Arabia: a cross-sectional study (Questionnaire), https://doi.org/10.6084/m9.figshare.30510647.v1. ^33^ This project contains following extended data
• Survey questionnaire, participant information and consent form.docxf Data are available under the terms of the Creative Commons Zero “No rights reserved” data waiver (CC BY 4.0 Public domain dedication).
