# Supplementary material for: Oral Health Knowledge, Attitude, And Practices Among Residents Of Al-kharj, Saudi Arabia: A Cross-sectional Study
Source: F1000Res. 2025 Nov 26;14:1315. [Version 1] doi: 10.12688/f1000research.172953.1 (PMC12822687; doi:10.12688/f1000research.172953.1)
Supplement: Peer review history

# Reviewer report: Reviewer response for version 1

**DOI:** 10.5256/f1000research.190723.r446248

**Published:** 2026-01-06

**Authors:** Laliytha Bijai Kumar

**Affiliations:** 1 King Saud bin Abdulaziz University for Health Sciences, Riyadh, Riyadh Province, Saudi Arabia

Abstract: Conclusion is too concise. Mention key findings correlating to the aim to conclude the abstract. 

 Discussion: The first paragraph could focus on the study's novelty and purpose rather than jumping right into interpreting the results. 

 References: Consider rearranging references to recent, relevant literature within the past decade. Older references may be obsolete in the current context unless they relate to definitions, terminology, classifications, etc.

Is the work clearly and accurately presented and does it cite the current literature?

Partly

If applicable, is the statistical analysis and its interpretation appropriate?

Yes

Are all the source data underlying the results available to ensure full reproducibility?

Yes

Is the study design appropriate and is the work technically sound?

Yes

Are the conclusions drawn adequately supported by the results?

Partly

Are sufficient details of methods and analysis provided to allow replication by others?

Yes

Reviewer Expertise:

Oral Medicine, Oral cancer, tobacco products, oral radiology, maxillofacial radiology

I confirm that I have read this submission and believe that I have an appropriate level of expertise to confirm that it is of an acceptable scientific standard, however I have significant reservations, as outlined above.

# Reviewer report: Reviewer response for version 1

**DOI:** 10.5256/f1000research.190723.r437163

**Published:** 2026-01-02

**Authors:** Harshkant Gharote

**Affiliations:** 1 Batterjee Medical College, Jeddah, Saudi Arabia

The discussion is generally well-structured and connects findings to existing literature, but it occasionally makes interpretative jumps not fully supported by data, particularly regarding the support of the authors' findings. Nonetheless, the authors have used studies on child polpulation to support their data. This can be robustly supported by the adult studies conducted in Arab coutries including Saudi Arabia. The discussion could better situate the findings within Saudi Arabia’s uniqueoral healthcare patterns.

 although, Social desirability bias is mentioned in discussion, authors can add a note in sampling technique in materials and methods to enhance readers' interests.

 The apostrophe symbol added to word behaviour should be removed.

Is the work clearly and accurately presented and does it cite the current literature?

Yes

If applicable, is the statistical analysis and its interpretation appropriate?

Yes

Are all the source data underlying the results available to ensure full reproducibility?

Yes

Is the study design appropriate and is the work technically sound?

Yes

Are the conclusions drawn adequately supported by the results?

Yes

Are sufficient details of methods and analysis provided to allow replication by others?

Yes

Reviewer Expertise:

Prevalence, medical management of oral elsions, radiographic presentation of oral and maxillofacial pathologies.

I confirm that I have read this submission and believe that I have an appropriate level of expertise to confirm that it is of an acceptable scientific standard.

## References

[1] : Gender Differences in Oral Health Knowledge and Practices Among Adults in Jeddah, Saudi Arabia. *Clinical, Cosmetic and Investigational Dentistry* .2022;Volume 14: 10.2147/CCIDE.S379171 235-244 10.2147/CCIDE.S379171 PMC935940235957700

[2] : Association of oral health awareness and practice of proper oral hygiene measures among Saudi population: a systematic review. *BMC Oral Health* .2023;23(1) : 10.1186/s12903-023-03522-w 10.1186/s12903-023-03522-w 37875884 PMC10594747

# Reviewer report: Reviewer response for version 1

**DOI:** 10.5256/f1000research.190723.r443015

**Published:** 2026-01-02

**Authors:** Mudita Chaturvedi

**Affiliations:** 1 Dr. D. Y. Patil Dental College and Hospital, Pimpri, Pune, India

Dear Authors, well performed research.....overall manuscript is good, but I have few queries - 

**Abstract & Results section** state: *Women exhibited significantly better knowledge than men* (p = 0.0001)

**Tables 3, 4, 5 and Table 6** clearly show: **Males have higher knowledge scores**

 Regression coefficient for *Male* is positive and significant (β = 0.56, p = 0.001)

**Discussion and Conclusion** again state: Women demonstrated superior knowledge and practices

 Please clarify this because it should be clearly stated so the readers will have a proper understanding. 

 Further, the methods and results are well presented. My suggestion is to recheck the overly long sentences and modify them to short and effective sentences. 

 Rewrite the aim properly -"

 Since dentists are key in educating communities, evaluating knowledge, attitudes, and behaviour’s at the population level is vital. This study investigates these factors among adults in Al-Kharj and examines the associations between demographic factors and oral health practices."

Is the work clearly and accurately presented and does it cite the current literature?

Yes

If applicable, is the statistical analysis and its interpretation appropriate?

Yes

Are all the source data underlying the results available to ensure full reproducibility?

Yes

Is the study design appropriate and is the work technically sound?

Yes

Are the conclusions drawn adequately supported by the results?

Yes

Are sufficient details of methods and analysis provided to allow replication by others?

Yes

Reviewer Expertise:

dentistry, dental education, oral health, dental survey..

I confirm that I have read this submission and believe that I have an appropriate level of expertise to confirm that it is of an acceptable scientific standard, however I have significant reservations, as outlined above.

# Reviewer report: Reviewer response for version 1

**DOI:** 10.5256/f1000research.190723.r443016

**Published:** 2026-01-02

**Authors:** Suresh Kandagal Veerabhadrappa

**Affiliations:** 1 SEGi University, Petaling Jaya, Selangor, Malaysia

1. **Misinterpretation of “knowledge about primary teeth”**

In **Results** and **Table 2**, you report:

- Only *28.6% recognized the importance of treating primary teeth*

But in **Table 2**, the item reads:

 “The tooth decay of milk teeth need to be treated” – **71.4% correct**

 This is a **direct contradiction**.

  

1. **Sampling method inconsistency**

You state:

- “Participants were recruited from shopping malls”
- Later: “shopping malls and public parks”
- Sampling described as **multistage stratified random sampling**

improve **methodological transparency**

  

1. Statistical concerns

Mention Fisher’s exact test wherever cells count is <5

  

1. The conclusion states:

“This population-based study…”

 Corrected it as community based study

  

1. Discussion repeats incorrect gender interpretation
2. **Ethics section duplicated**

Ethics and consent appear **twice**.

  

1. Regression table (6)

•  Confidence intervals for education are inconsistent (lower = –0.11, upper = 1.01 but p = 0.790)

 •  Education coefficient is negative but interpreted as non-significant → fine, but interpretation should be explicit.

 Correct this

Is the work clearly and accurately presented and does it cite the current literature?

Yes

If applicable, is the statistical analysis and its interpretation appropriate?

Yes

Are all the source data underlying the results available to ensure full reproducibility?

Yes

Is the study design appropriate and is the work technically sound?

Yes

Are the conclusions drawn adequately supported by the results?

Yes

Are sufficient details of methods and analysis provided to allow replication by others?

Yes

Reviewer Expertise:

Orofacial pain, TMDs, CBCT, Intraoral radiographic techniques

I confirm that I have read this submission and believe that I have an appropriate level of expertise to confirm that it is of an acceptable scientific standard.
